# Androgen/Androgen Receptor Signaling in Ovarian Cancer: Molecular Regulation and Therapeutic Potentials

**DOI:** 10.3390/ijms22147748

**Published:** 2021-07-20

**Authors:** Wei-Min Chung, Lumin Chen, Wei-Chun Chang, Sheng-Yuan Su, Yao-Ching Hung, Wen-Lung Ma

**Affiliations:** 1 Department of Obstetrics and Gynecology, China Medical University Hsinchu Hospital, Hsinchu 30272, Taiwan; qqrice68@yahoo.com.tw (W.-M.C.); silviachen6585@gmail.com (L.C.); deeppond2@gmail.com (S.-Y.S.); 2 Sex Hormone Research Center, Department of Obstetrics and Gynecology, China Medical University Hospital, Taichung 40403, Taiwan; wei66@iris.seed.net.tw; 3 Department of Obstetrics and Gynecology, Asia University Hospital, Taichung 41354, Taiwan; 4 Graduate Institute of Biomedical Sciences, School of Medicine, China Medical University, Taichung 40403, Taiwan; 5 Department of Nursing, Asia University, Taichung 41354, Taiwan

**Keywords:** androgen receptor, ovarian cancer, cancer stem/progenitor cells, microRNA

## Abstract

Ovarian cancer (OVCA) arises from three cellular origins, namely surface epithelial cells, germ cells, and stromal cells. More than 85% of OVCAs are EOCs (epithelial ovarian carcinomas), which are the most lethal gynecological malignancies. Cancer stem/progenitor cells (CSPCs) are considered to be cancer promoters due to their capacity for unlimited self-renewal and drug resistance. Androgen receptor (AR) belongs to the nuclear receptor superfamily and can be activated through binding to its ligand androgens. Studies have reported an association between AR expression and EOC carcinogenesis, and AR is suggested to be involved in proliferation, migration/invasion, and stemness. In addition, alternative AR activating signals, including both ligand-dependent and ligand-independent, are involved in OVCA progression. Although some clinical trials have previously been conducted to evaluate the effects of anti-androgens in EOC, no significant results have been reported. In contrast, experimental studies evaluating the effects of anti-androgen or anti-AR reagents in AR-expressing EOC models have demonstrated positive results for suppressing disease progression. Since AR is involved in complex signaling pathways and may be expressed at various levels in OVCA, the aim of this article was to provide an overview of current studies and perspectives regarding the relevance of androgen/AR roles in OVCA.

## 1. Introduction

Ovarian cancer (OVCA) is among the most lethal gynecological malignancies and has a variety of cellular origins, histological characteristics, and therapeutic responses [1]. Despite OVCA comprising only 3% of all cancer incidents, OVCA has an extremely high mortality rate, representing the fifth leading cause of cancer-associated death in women [2,3,4]. In 2018, approximately 14,000 OVCA-related deaths were reported in the US, accounting for 5% of all cancer deaths in women [5]. Several epidemiological factors are thought to contribute to OVCA development, including hereditary factors, such as family history, the presence of mutations in the breast-cancer-associated genes *BRCA1* and *BRCA2*, and age; estrogenic influences, such as early menarche and menopause after 52 years of age; environmental risk factors; and lifestyle choices, such as exposure to polycyclic aromatic hydrocarbons (PAH), cigarette smoking, and obesity [6].

Androgen receptor (AR) was well-characterized as ligand-dependent transcription factor, which belongs to the nuclear receptor superfamily [7]. Androgen, a steroid hormone, has been suggested to play critical roles in male sexual development and reproductive function and the maintenance of the male phenotype. Androgens are also well-identified ligands for AR. Moreover, 5α-reductase converts intracellular testosterone into 5α-dihydrotestosterone (DHT), which has a high affinity for AR. Interactions between androgen and AR promote the dissociation of AR from heat shock proteins, allowing AR to translocate into the nucleus and bind androgen response elements (AREs) on specific non-promoter distal enhancer regions and then recruited coactivators to activating target gene expression [8,9,10]. This gene transcriptional mechanism is commonly referred to as “classical transactivation” or “ligand-dependent transactivation”.

AR has also been reported to become activated and translocate into the nucleus without binding to a ligand, which is referred to as “non-classical transactivation” or “ligand-independent transactivation” [11,12,13,14]. Non-classical AR signaling is known to involve mitogen-activated protein kinase (MAPK)/extracellular signal-regulated kinase (ERK) activation; the activation of mammalian target of rapamycin (mTOR) through the phosphoinositide 3-kinase (PI3K)/protein kinase B (Akt) pathway; and plasma membrane components, including G-protein-coupled receptors (GPCRs) and sex-hormone-binding globulin receptor (SHBGR), which modulate intracellular Ca^2+^ concentration and cyclic adenosine monophosphate (cAMP) levels, respectively [15,16,17].

In cancer biological research, AR has been reported to be expressed in many cell types, and androgen/AR signaling has been found to promote tumorigenesis and metastasis in several cancer types, including OCVA [18,19]. Accumulating evidence consistently supports AR overexpression in OVCA. Immunohistochemistry studies have shown that AR expression is detected in approximately 43.5–86% of epithelial OVCA (EOC) [20,21,22]. These findings suggest that AR plays a pivotal role in OVCA progression and might represent a potential therapeutic target for OVCA treatment. Therefore, this review aims to evaluate the current knowledge associated with androgen/AR signaling in the pathological progression of OVCA.

## 2. AR Expression, Genetic Polymorphisms, Function, and Regulation in Human OVCA

### 2.1. General Description, Classification, and Pathological Features of OVCA

Sex-cord/stromal tumors develop from the stroma or the sex cord. The stroma and sex cord are tissues that support the ovary and produce the female hormones estrogen and progesterone. Stromal tumors are a rare tumor type, comprising less than 7% of all OVCAs. Sex-cord/stromal tumors can be identified at any age but most commonly affect young women (median age 27 years), causing abnormal vaginal bleeding and pain. Surgery is the primary treatment option for stromal tumors, and the prognosis is generally favorable [23,24]. Although it is a rare subset of malignant ovarian tumors, Alexiadis et al. (2011) profiled human nuclear receptors, including AR, in two GCT-derived (granulosa cell tumors (GCTs) that arise from the stromal cells of the ovary) cell lines, COV434 and KGN. Moreover, their data showed that AR is the most abundant of the steroid receptors in the GCT, but the role and functions of AR are still unclear in this study [25].

Ovarian teratoma (OVTC) arises from germ cells and is typically benign. Malignant teratoma, known as teratocarcinoma, is a minority tumor type (less than 10%) of the OVTC [26]. OVTC may comprise various tissues, including glandular tissues, multilayered epithelial tissues, hair follicles, bone, teeth, and cartilage, and it occurs in all ages. The development of OVTC has been attributed to the aberrant meiosis of germinal cells within the ovary; however, the detailed pathogenesis underlying this tumor type remains poorly understood [27,28,29,30,31]. OVTC is caused by the abnormal development of pluripotent germ or embryonic cells, making it a good model for studying the behavior of cancer stem/progenitor cells (CSPCs) [32].

More than 85% of OVCA are EOCs, which are the most lethal gynecological malignancies that arise from ovarian surface epithelial cells (OSECs) [1]. The four major histological EOC subtypes include serous carcinoma (SC), mucinous carcinoma (MC), endometrioid carcinoma (EC), and clear cell carcinoma (CCC), which can be identified by cancer cell morphology [33,34]. The EOC subtypes differ in etiology, malignancy, response to chemotherapy, and prognosis according to characteristics, including histopathology, immunohistochemistry, and molecular genetics [35,36,37]. Generally, high-grade SC (HGSC) describes histologically aggressive, highly proliferative, and *p53* mutation-associated, with high *p16* expression and the loss of *BRCA1* expression. HGSC is often initially chemosensitive, followed by the development of resistance and poor prognosis, whereas low-grade SC (LGSC) is associated with a noninvasive, serum-confined, less proliferative, and *KRAS* and *BRAF* mutation-associated, with generally intermediate chemosensitivity, except metastasis [38,39,40]. In Taiwan, MC is second populated primary EOC subtype [33,38]. MC has been reported that accompanied with *KRAS* mutation (43.6%) and HER2 overexpression (18.8%) [38,41,42]. It has been reported that MC is sensitive to chemotherapy with favorable prognosis [38]. EC (Endometrial subtype EOC) is often considered to represent a secondary neoplasm that arises from endometriosis or a pre-existing borderline adenofibroma [1,43]. It accounts for about 10% of EOCs and occurs most frequently in women of peri-menopausal age, and most are found at an early stage [38]. Molecular studies have shown that hereditary nonpolyposis colorectal carcinoma is an EC risk factor, and mutations in *CTNNB* (β-catenin), *PI3CA* (PI3K), and *PTEN* (phosphatase and tensin homolog) have also been reported to be EC risks. Similar to LGSC, EC is highly sensitive to chemotherapeutic agents and associated with better prognosis in patients [37,38]. The fourth subtype, CCC, is associated with atypical endometriosis, occurring with an incidence approximately equal to that of EC among women in Taiwan [33,38]. Genetic analysis showed that CCC always bears mutations in *ARID1A* genes and upregulation of HNF-1 protein [38]. Recent clinical studies have shown that CCC exhibits poor prognosis and high levels of resistance to chemotherapy agents, as well as being associated with high mortality [38].

Although EOC represents a heterogeneous disease, decisions regarding standard therapeutic strategies continue to rely on the diagnosed stage and grade rather than histological type [44]. The current treatment for newly diagnosed EOC is surgical bulk reduction, followed by adjuvant chemotherapy [45]. Only 20–30% of EOC patients are typically diagnosed at an early stage, when the neoplasm bulk removal is curative. The majority of patients have already progressed to an advanced stage when first diagnosed with EOC [46]. For early stage EOC, appropriate surgery and chemotherapy can increase patient survival rates up to 90–95% [47]. However, most advanced-stage patients have lower survival rates (<30%) during the 5-year period after treatment with surgery and chemotherapy [48]. The first-line chemotherapy option for treating advanced-stage EOC is the combination of platinum- and taxan-based adjuvant chemotherapies, to which approximately 80% of women are responsive [45,49,50,51]. Unfortunately, 70–80% of advanced-stage EOC patients relapse after treatment, with particularly high relapse rates observed for SC (up to 70%) [48,52]. Therefore, these therapeutic strategies for EOC remain controversial.

Some factors had been associated with poor prognosis of EOC, including (1) complicated personal risk factors, such as individual diet, inherited gene mutations, and ages; (2) a lack of early diagnosis markers; and (3) resistance to chemotherapy. Thus far, several studies have focused on the relationships between EOC treatments and these factors, both in vivo and in vitro.

### 2.2. Biochemical Functions of Androgen/AR Signaling (Classical vs. Non-Classical Androgen/AR Signaling)

The AR protein structure consists of three major functional domains: N-terminal domain (NTD), DNA-binding domain, and ligand-binding domain. The NTD exerts a modulatory function during protein–protein interactions, such as those with coactivators or other transcription factors [18]. The DNA-binding domain is a cysteine-rich region consisting of two zinc finger structures. These structures are responsible for AR–DNA interactions, which regulate gene expression through binding to AREs at distal enhancer regions of target genes [9,10]. The ligand-binding domain provides a site for AR–ligand binding, which activates AR translocation activity. Classical action of AR is indicated as androgen acts through binding with AR. The binding of androgen to the AR results in the dissociation of heat-shock proteins, followed by the translocation of the AR/androgen complex into the nucleus, where it binds AREs on the DNA [40]. In addition to classical androgen/AR activation, AR has been documented to function in a rapid, transient activation mode without binding to a ligand, known as “non-classical action” [11,12,13,14]. The non-classical AR signaling is known to involve MAPK/ERK activation, mTOR activation via the PI3K/Akt pathway, and the involvement of plasma membrane components, including GPCRs and the SHBGR, which modulate intracellular Ca^2+^ concentrations and cAMP levels [12,16,17]. In addition, another form of AR activation has been observed without ligand binding [15,16], and these ligand-independent pathways may be correlated with AR phosphorylation or AR-associated signaling proteins [16,17].

### 2.3. Associations of Androgen Levels, Gene Polymorphisms, and AR Expressions with OVCA Risks

EOC has been shown to be an androgen-responsive tissue [21,53]. Androgen concentration associated with risk of OVCA has been reported during earlier clinical studies [54,55]. Cuzick et al. (1983) assessed urinary concentrations of DHEA (dehydroepiandrosterone, a steroid hormone that serves as a precursor to androgen), androsterone, and etiocholanolone in 12 patients who developed OVCA from among 1484 total patient samples. They found that the DHEA, androsterone, and etiocholanolone concentrations were lower in the 12 OVCA patients when compared with those in an age-matched group [54]. Another study, performed by Helzlsouer et al. (1995), compared the serum levels of adrenal androgens in 31 patients with OVCA with those in 62 control women matched for race, age, and menopausal status. The levels of androstenedione (4.5 ± 2.8 versus 3.3 ± 2.1 nmol/L; *P* = 0.03) were significantly higher in the cancer patients than in the control subjects [55]. The blood concentrations of testosterone have also been assessed, which indicated that free testosterone concentrations were associated with the risk of OVCA (odds ratio (OR) = 0.45, 95% confidence interval (CI) = 0.24–0.86, *P* = 0.01), specifically SC (OR = 0.90, 95% CI = 0.75–1.08, *P* = 0.02), in postmenopausal women [7,56,57] These findings suggested the involvement of androgen in modulating the development of OVCA pathogenesis, especially in premenopausal women who had differences in the levels of DHEA (23.9 ± 15.6 versus 11.4 ± 5.9 nmol/L; *P* = 0.02) and androstenedione (4.9 ± 2.8 versus 3.4 ± 1.7 nmol/L; *P* = 0.05), was observed in premenopausal women (n = 13), but not in postmenopausal women (n = 18).) [7].

Studies showed that AR expression varied across histological EOC subtypes. Recent studies showed that AR is expressed in all EOC subtypes, but higher expression levels were observed in SCs than in other EOCs [20,22]. This observation was supported by the study by Lee et al. (2005), who reported that AR was expressed in 43.7% of primary OVCA samples, and the highest percentages of AR expression were observed in SC (47.5%) samples of total cases [14]. Sheach et al. (2009) reported that AR expression scores by immunohistochemistry showed no correlation with the International Federation of Gynecology and Obstetrics (FIGO) stage, residual disease, or preoperative cancer antigen 125 (CA125) levels, whereas AR expression can be detected specifically in EOC serous subtype [21]. Similarly, a study by de Toledo et al. (2014) found that AR expression tended to be more prevalent in serous than in non-serous tumors [58]. Furthermore, studies have shown that the AR-positive SC subtype promotes cancer development through the regulation of the cell cycle by androgen stimulation [53,59,60]. These studies have implied that AR could serve as a therapeutic target in OVCA.

AR is the conventional cellular receiver for androgens. The AR gene is composed of eight exons. Exon 1 contains two polymorphic trinucleotide repeats: a 9–39 CAG repeat (polyglutamine, polyQ) and a 14–27 GGN repeat (polyglycine, polyG) [18,61]. Many studies have focused on AR gene polymorphisms, particularly the association between the polyQ repeat and OVCA risk; however, this association remains controversial [62,63,64,65,66,67,68,69]. Engehausen et al. (2000) evaluated the human *AR* (h*AR)* mutations in 38 human OVCA cell lines associated with different AR expression patterns, and the results showed no mutations in the h*AR* gene. They concluded that mutation screening of h*AR* might not provide any information for OVCA risk assessment [70].

## 3. Androgen/AR Signaling in OVCA Experimental Models

### 3.1. Androgen/AR Signaling Function in the Gynecological System

Accumulated studies have indicated that androgen or AR activity can affect OVCA progression and could serve as potential therapeutic targets for the treatment of this disease. Syed et al. (2001) found that testosterone and DHT significantly stimulated cell growth in both malignant and normal ovarian cell lines [71]. This androgen-stimulated growth could be reversed by co-treatment with the anti-androgen 4-hydroxyflutamide [71]. Edmondson et al. (2002) demonstrated that the ovarian surface epithelium is an androgen-responsive tissue and that androgen treatment increased proliferation and decrease cell death in eight primary cultures of human ovarian surface epithelium cells [72]. Studies have also shown that androgen/AR activity was associated with EOC proliferation and migration/invasion [61,73]. The potential mechanism is that androgen downregulates expression of transforming growth factor-β (TGF-β) receptor, a potent suppressor of epithelial cell growth, to promote OVCA cell growth [73,74]. The epidermal growth factor receptor (EGFR) is overexpressed in 30–98% of EOCs, and the activation of EGFR-associated signaling cascades has been linked to cell proliferation, migration, and invasion; angiogenesis; and resistance to cell apoptosis [75].

Androgen-induced EOC proliferation may be partially mediated by enhanced interleukin (IL)-6 and IL-8 expression, which might promote EOC growth via the activation of the AR gene promoter [76]. Ligr et al. (2011) tested the effects of androgen treatment on cell invasion in OVCAR-3 and SKOV-3 cell lines, using an in vitro Matrigel invasion assay, and observed significantly increased invasiveness in cells treated with synthetic androgen compared with hormone-free cells [77]. Du et al. (2016) identified the moderately aggressive OVCA cells are associated with the overexpression of AR, which is associated with increased cell migration and invasion, promoting a more aggressive OVCA phenotype [78]. In a study, Zhu et al. (2016) established AR overexpressing OVCAR-3 and SKOV-3 OVCA cell lines and evaluated their effects on proliferation and migration in vitro. AR overexpression promoted proliferation and migration in both OVCA cell lines, as determined by 3-(4, 5-dimethylthiazol-2-yl)-2, 5-diphenyltetrazolium bromide (MTT) proliferation, and transwell migration assays [67]. Together, these findings indicated that AR associated with EOC progression.

### 3.2. Androgen/AR Signaling in OVCA Stemness

CSPCs are thought to be responsible for cancer phenotypes, including pathogenesis, metabolism, metastasis, drug resistance, and relapse [79]. Previous studies have identified CSPCs among primary OVCA cells, and some OVCA cell lines have been reported to exhibit rare populations of potential cancer stem cells, as identified by specific CSPCs markers, with unique self-renewal capacity and resistance to chemotherapy [80,81]. Studies have shown that several glycoproteins, including CD133, CD117, CD24, CD44, aldehyde dehydrogenase 1 (ALDH1), and ATP binding cassette subfamily G member 2 (ABCG2/BCRP), can be used as CSPC markers in ovarian tissue [82].

Numerous studies have demonstrated that AR regulates progression of cancer stem cell in various cancer types, including OVCA [83]. A study conducted by Chung et al. (2014), using OVTC cells, provided evidence that ligand-independent AR functions in CSPCs (e.g., CD133^+^ cells) facilitated OVTC cell growth [83]. Chen et al. (2014) examined EC cells and showed that AR expression facilitated CSPC progression and the development of cisplatin resistance in EC cells [84]. Lin et al. (2018) showed that Nanog expression correlated with AR expression and Nanog promoter transcription can be activated under androgen treatment, which resulted in OVCA cells proliferation, migration, sphere formation, and colony formation. Based on their findings, the authors indicated that interaction of Nanog with AR signaling axis promotes ovarian CSCs characteristics under androgen treatment [85]. These studies showed that AR expression promotes CSPC self-renewal through both classical and non-classical signaling pathways. Although previous reports have documented the potential involvement of AR in cell stemness, little is known regarding the contribution of AR to EOC subtypes.

Chemoresistance remains a major challenge to cancer chemotherapy. Most patients respond to chemotherapies during the initial treatment period combined with surgery; however, many patients exhibit a limited response to chemoreagents and become resistant to subsequent treatments [44,50]. According to previous studies, CSPCs presented higher expression and activity of ABC transporters, which are the membrane proteins thought to be responsible for multidrug resistance (MDR). MDR refers to those mechanisms through which many cancers develop resistance to chemotherapy drugs, which are major factors in the failure of various chemotherapy regimens [86]. ABC transporter proteins are transmembrane proteins involved in drug efflux that utilize energy-consuming ATP hydrolysis to export drugs out of cells [87]. They can be divided into ABCB, ABCC, and ABCG families and play important roles in drug resistance [88]. Some ABC transporters have been associated with paclitaxel transport, including ABCB1, ABCC2, and ABCG2 [89,90,91]. Therefore, cancer cells that express higher levels of ABC transporters are more prone to drug resistance [89,90,91].

In addition to the role played by AR in OVTC CSPCs, Chung et al. (2019) also showed that AR regulated the expression of ABCG2, an MDR-associated membrane protein, in SC cells under paclitaxel treatment conditions through ligand-independent AR translocation activity. Furthermore, treatment with ASC-J9, an AR degradation enhancer, eliminated the paclitaxel–AR–ABCG2 axis and enhanced drug sensitivity in OVCA cell lines. Chung et al. showed that targeting the degradation of AR protein is more beneficial than anti-androgen to treating AR^+^ OVCA [92].

## 4. Current Clinical Trials of Targeting Androgen/AR Therapy in OVCA

### 4.1. Androgen Ablation Therapy in OVCA

AR has been reported to be differentially expressed across EOC subtypes [20,22], and studies have shown that androgen/AR activity is associated with OVCA progression in vivo and in vitro [77,78,93]. Therefore, various anti-androgen pharmacological agents have been developed as a therapeutic approach for OVCA treatment. Many clinical androgen ablation applications have been expanded to the treatment of women diagnosed with OVCAs, including flutamide (a nonsteroidal drug with anti-androgen properties), bicalutamide (an oral nonsteroidal anti-androgen), and goserelin (a gonadotropin-releasing hormone agonist that eventually decreased androgen secretion by suppressing the release of follicle-stimulating hormone from the pituitary gland). These anti-androgen drugs have been widely used to treat men with prostate cancer, without severe adverse effects [94].

The early clinical reports examining the effects of flutamide, bicalutamide, and goserelin have been conducted in OVCA patients. The phase II clinical trial examining the use of flutamide in 68 EOC patients treated with platinum-based chemotherapy was reported by Tumolo et al. (1994) [95]. In this study, 32 patients completed oral flutamide treatment (750 mg/day) for at least 2 months, among which two patients responded to treatment (one complete response lasted for 44 weeks, and one partial response lasted for 72 weeks), and nine patients had stable disease for a median of 24 weeks (range: 12–48 weeks). The authors suggested that flutamide treatment in EOC patients with chemotherapy pretreatment was invalid and associated with side effects. Another phase II study of flutamide (300 mg/day) in high-grade OVCA patients (stages III and IV) was reported by Vassilomanolakis et al. (1997) [96]. The results showed that partial response and disease stabilization were observed in one case (4.3%) that lasted for 3 months and two cases (8.7%) that lasted for 7 to 8 months among the 23 patients examined.

More recently, a phase II study of bicalutamide (50 mg orally daily) and goserelin (3.6 mg subcutaneously every 4 weeks) in stages III and IV EOC patients was reported by Levine et al. (2007) [97]. A total of 35 patients were enrolled, and the progression-free survival (PFS) among patients receiving the protocol therapy during second disease remission (21 patients) was 11.4 months (95% CI, 10.2–12.6 months). The PFS for patients receiving protocol therapy during their third or fourth disease remission (11 patients) was 11.9 months (95% CI, 10.8–14.1 months). This report demonstrated that the use of goserelin and bicalutamide did not appear to prolong PFS in patients with EOC who have experienced two or more complete disease remissions.

According to these clinical trials, only a few EOC patients were responsive to anti-androgen therapy combined with other chemotherapies. However, the results of anti-androgens (flutamide, bicalutamide, and goserelin) that block the androgen/AR signaling axis in clinical trials have been controversial, and these treatments have been ineffective for curing most patients of this difficult-to-treat disease [98]. One possible explanation for the failure of anti-androgen therapy efficacy might be associated with insufficient patient numbers, heterogeneity among patient demographics or disease characteristics, and pathological tumor characteristics [98,99,100]. However, none of these controversial clinical trials performed any clear dissection of the role played by AR in EOC subtypes, which likely contributed to the failure to obtain any encouraging clinical results.

### 4.2. AR Degradation Therapy for OVCA

An AR degradation enhancer, dimethylcurcumin (ASC-J9), has also demonstrated effective tumor inhibition, including against OVCA. Lin et al. (2018) showed that ASC-J9 inhibitor promoted EOC cell proliferation, migration, and sphere formation in CSPCs in vitro and in vivo [85]. Thus far, although bench studies and clinical trials of various androgen/AR axis antagonists and AR degradation enhancers have been studied, the results remain controversial relative to the treatment of EOC. AR as a target for EOC therapy remains a critical issue in the field that requires further evaluation. A number of clinical studies (Table 1) and in vitro studies (Table 2) have addressed the anti-androgen or anti-AR therapies in EOC patients.

### 4.3. Potential for the Use of Anti-Androgen/AR and Anti-PARP Combination Therapy for OVCA

Poly (ADP-ribose) polymerase (PARP) inhibitors (PARPi) represent the first FDA-approved treatment to apply the concept of synthetic lethality (cell death induced by single strain break) to the treatment of EOC. Although only 10–15% of patients with high-grade serous OVCA (HGSOC) harbor germline *BRCA1* or *BRCA2* mutations, approximately 50% of patients are diagnosed with tumors that exhibit homologous recombination (HR) deficiencies due to either a germline or somatic mutation in the HR system [101]. The results of a phase II study showed that patients with germline *BRCA1/2*-mutated advanced OVCA who had received three or more chemotherapies had a median PFS of 7 months, a median overall survival (OS) of 16.6 months, and a 1-year OS rate of 64.4% [102]. Studies of olaparib to treat women with HGSOC recurrence reported that patients who received olaparib maintenance treatment showed a longer PFS (median, 8.4 vs. 4.8 months; hazard ratio, 0.35; 95% confidence interval (CI), 0.25–0.49; *P* < 0.001) [103,104].

Several studies have reported that treatment with the PARPi olaparib, combined with enzalutamide and olaparib, demonstrated synergistic effects in prostate cells and orthotopic xenograft models [105,106]. In both AR-responsive and AR-independent cell lines, the use of enzalutamide (Xtandi and Astellas) reduced the expression of HR genes, including *BRCA1* [106,107]. In our findings, we indicated that targeting AR eliminated ABCG2-associated drug resistance, and treatment with the AR degradation enhancer (ASC-J9) provided a synergistic effect with the first-line chemotherapeutic agent paclitaxel in SC [92]. Thus, in addition to advocating for further testing of the combination of paclitaxel and AR inhibitor, targeting AR combined with PARPi may remedy the deficiencies of PARPi treatment and provide an advanced therapeutic strategy for SC.

## 5. Conclusions

**Figure 1:**
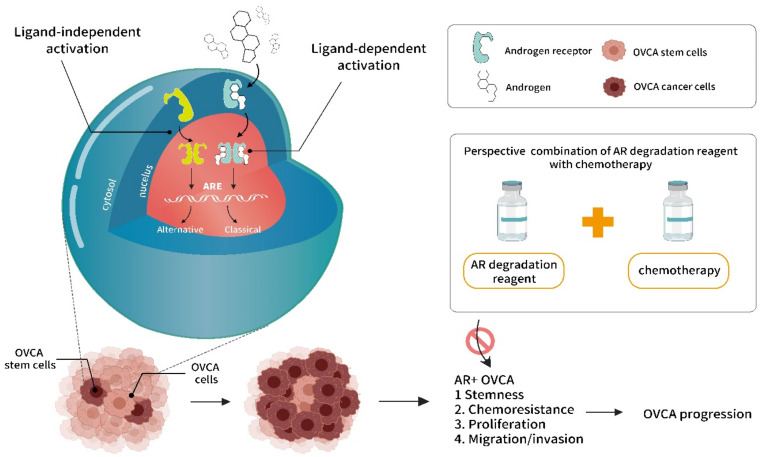
Illustration of androgen/androgen receptor (AR) signaling in ovarian cancer (OVCA) progression, and potential benefit of targeting AR for OVCA therapeutics. The androgen/AR signaling, including ligand dependent vs. independent activation modes that bind on androgen response elements (AREs; including classical and alternative) on promoter region for target gene expressions. The unique androgen/AR signaling occurring at the cancer stem/progenitor cells (CSPCs) of OVCA, which enriches CSPC population. Consequently, lead to enriched cancer stemness, chemoresistance, fast growth of cells, and increase mobility of OVCA. The proposed therapeutics on targeting AR degradation combination with conventional chemotherapy might acquire therapeutic benefit to improve OVCA progression via suppressing AR+ OVCA cells.

Although the role played by AR in OVCA has been studied in vitro, providing additional understanding regarding this relationship, the development of an effective in vivo model for AR research and combinational therapy for use in preclinical trials for this disease remains an active and increasingly popular area of investigation. To better understand the mechanism of AR signaling and to design proper therapies against AR in OVCA, an increased focus must be placed on identifying the AR activation mechanisms and target genes that contribute to tumor recurrence, and the development of therapy resistance is necessary. Advancements in this mechanistic understanding will shed light on potentially effective combination therapies for patients with the AR^+^ OVCA subtype. Discerning the intricacies and crosstalk between AR and the tumor microenvironment may also provide advantages for OVCA treatment and would not only advance our understanding of the role played by AR in cancer progression but also identify new treatment strategies through which AR signaling can be blocked to improve outcomes for women with OVCA. A new perspective on combination therapy of AR degradation and chemoreagents is illustrated in Figure 1.

## Figures and Tables

**Table 1:** Clinical trials of Androgen/ androgen receptor inhibitors in the treatment of ovarian cancer patients.

Study	No. of Patients	Clinical Trial	Treatment	Key Findings
Thompson et al. (1991)	62	NR	cyproterone acetate	6.8% patients partially responded.
Tumolo et al. (1994)	32/68	phase II	oral flutamide treatment (750 mg/day)	Combined chemotherapy was invalid with side effects.
Van der Vange et al. (1995)	33	Phase II	flutamide (500 mg i.m)	1 patients showed stable disease for 8 months.
Vassilomanolakis et al. (1997)	43	phase II	flutamide (300 mg/day)	Partial response (4.3%) and disease stabilization (8.7%) in 23 patients.
Levine et al. (2007)	35	phase II	bicalutamide (50 mg/daily) and goserelin (3.6 mg/mon)	Failed to prolong PFS in EOC patients
Rachel et al. (2017)	59	phase II	Enzalutamide (160mg by mouth QD)	Currently recruiting patients (NCT01974765)
Susana et al. (2020)	42	Phase II	abiraterone (1000mg daily)	Closured.

Abbreviations: NR: not reported; AR: androgen receptor; EOC: epithelial ovarian cancer; PFS: progression free survival; QD: once daily; i.m: intramuscular.

**Table 2:** Current studies of targeting on androgen/ androgen receptor in ovarian cancer cells.

Study	Cell Lines	Treatment	MOA	Key Findings
Park et al. (2016)	OVCAR-3	Enzalutamide	NR	Showed efficacy in the ovarian cancer with AR expression.
Lin et al. (2018)	A2780 and SKOV3	ASC-J9	Suppressing AR→Nanog axis	AR promotes Nanog expression which contribute to CSPCs stemness in EOC cells
Chung et al. (2019)	HeyA8, SKOV3ip1, OVCAR-3	Paclitaxel/ASC-J9	Suppressing AR→ABCG2 axis	Degradation of AR is effective in suppressing OCSO subtype.
Addie et al. (2019)	A2780, OV90, OVCAR3, OVCAR8, SKOV3, COV362.4	Metformin/enzalutamide	AR→PI3K pathway	Anti-androgen failed to suppress AR+ EOC cells, implicating an ligand-independent pathway of AR.

Abbreviations: NR: not reported; AR: androgen receptor; CSPCs: cancer stem/progenitor cells; EOC: epithelial ovarian cancer; OCSC: ovarian cancer serous carcinoma; MOA: mode of action.

## Data Availability

Not applicable.
